# CT acquisition parameter selection in the real world: impacts on radiation dose and variation amongst 155 institutions

**DOI:** 10.1007/s00330-023-10161-w

**Published:** 2023-08-30

**Authors:** Yifei Wang, Philip Chu, Timothy P. Szczykutowicz, Carly Stewart, Rebecca Smith-Bindman

**Affiliations:** 1 Department of Epidemiology and Biostatistics, University of California San Francisco, 550 16Th Street, San Francisco, CA 94158 USA; 2 Departments of Radiology, Medical Physics, and Biomedical Engineering, University of Wisconsin-Madison, Madison, WI USA; 3 Department of Obstetrics, Gynecology and Reproductive Sciences, University of California, San Francisco, CA USA; 4 Philip R Lee Institute for Health Policy Studies, University of California San Francisco, 3333 California St, San Francisco, CA 94118 USA

**Keywords:** Tomography, X-ray computed, Radiation dosage, Dose–response relationship, Radiation

## Abstract

**Objective:**

Quantify the relationship between CT acquisition parameters and radiation dose, how often parameters are adjusted in real-world practice, and their degree of contribution to real-world dose distribution. Identify discrepancies between parameters that are impactful in theory and impactful in practice.

**Methods:**

This study analyses 1.3 million consecutive adult routine abdomen exams performed between November 2015 and Jan 2021 included in the University of California, San Francisco International CT Dose Registry of 155 institutions. We calculated geometric standard deviation (gSD) for five parameters (kV, mAs, spiral pitch, number of phases, scan length) to assess variation in practice. A Gaussian mixed regression model was performed to predict the radiation dose-length product (DLP) using the parameters. Three conceptualizations of “impact” were computed for each parameter. To reflect the theoretical impact, we predict the increase in DLP per 10% (and 15%) increase in the parameter. To reflect the real-world practical impact, we predict the increase in DLP per gSD increase in the parameter.

**Results:**

Among studied examinations, mAs, number of phases, and scan length were frequently manipulated (gSD 1.52–1.70); kV was rarely manipulated (gSD 1.07). Theoretically, kV is the most impactful parameter (29% increase in DLP per 10% increase in kV, versus 5–9% increase for other parameters). In real-world practice, kV is less impactful; for each gSD increase in kV, the DLP increases by 20%, versus 22–69% for other parameters.

**Conclusion:**

Despite the potential impact of kV on radiation dose, this parameter is rarely manipulated in common practice and this potential remains untapped.

**Clinical relevance statement:**

CT beam energy (kV) modulation has the potential to strongly reduce radiation over-dosage to the patient, theoretically more so than similar degrees of modulation in other CT acquisition parameters. Despite this, beam energy modulation rarely occurs in practice, leaving its potential untapped.

**Key Points:**

*• The relationship between CT acquisition parameter selection and radiation dose roughly coincided with established theoretical understanding.*

*• CT acquisition parameters differ from each other in frequency and magnitude of manipulation, with beam energy (kV) being rarely manipulated.*

*• Beam energy (kV) has the potential to substantially impact radiation dose, but because it is rarely manipulated, it is the least impactful CT acquisition parameter affecting radiation dose in practice.*

**Supplementary information:**

The online version contains supplementary material available at 10.1007/s00330-023-10161-w.

## Introduction

As a diagnostic tool, computed tomography (CT) imaging has expanded in use exponentially since its development in the 1970s. This expansion has improved the speed and accuracy of medical diagnosis. However, considerable variation persists in how CTs are performed across providers [1–4]. This means patients are exposed to very different amounts of radiation depending on where they obtain their CT examination. While some of the observed variation in radiation dose is attributable to differences in the make and model of CT scanners, or to differences in patient case mix (e.g., clinical indication for scanning, patient size, sex, and age), the dominant contributor to dose variation across imaging centers and hospitals is the imaging provider’s choice of protocol and associated CT acquisition parameters [3]. This remaining variation in performance is substantial, and ultimately inconsistent with the “as low as reasonably achievable” (ALARA) principle, a principle foundational to dose management, meant to limit iatrogenic harm, as ionizing radiation may increase cancer risks [5, 6].

Given a specific set of acquisition parameters, CT manufacturers and the medical physics community can predict the output from a CT scanner, and provide an estimate of dose (e.g., volume CT dose index [CTDI_vol_] or dose-length product [DLP]) prior to a patient being scanned. Technologists can see the expected dose prior to beginning a scan and can make adjustments if needed [7]. The scanner uses lookup tables to predict the dose based on selected settings including the scan range, mA profiles, beam energy, bowtie filter size, rotation time, scan mode (e.g., axial, cine, helical/spiral, and accompanying parameters like helical pitch), and beam collimation. Each of these adjustable parameters may or may not be altered on a patient-to-patient basis to fine-tune CT protocols to specific patient sizes and clinical indications. The degree of fine-tuning varies across radiology departments; some departments allow tremendous autonomy to technologists whereas others allow no autonomy to deviate from allowable protocols [8].

**Fig. 1:**
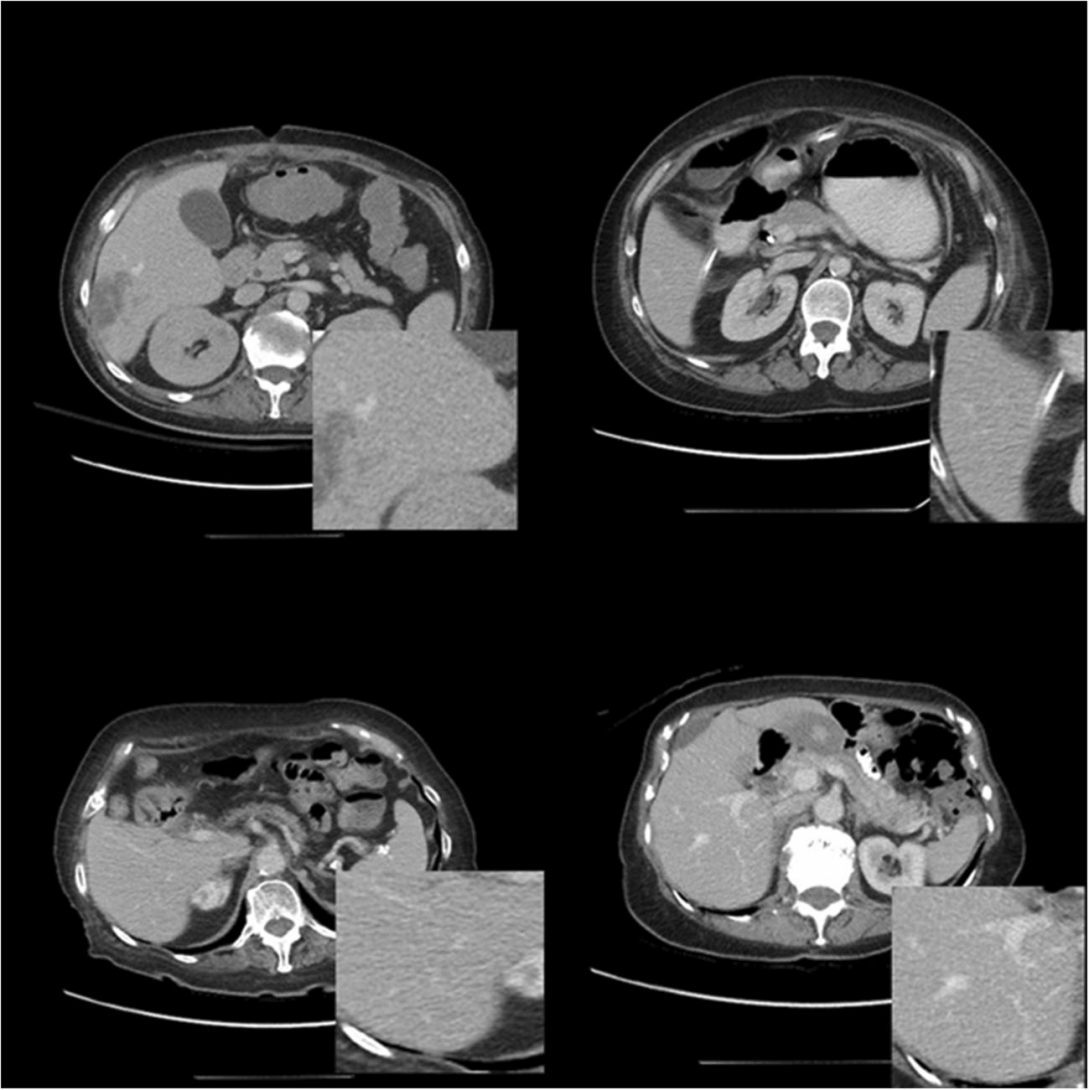
Abdominal CT images of different real-life patients, each effective diameter 310 mm. The figure depicts images with varying levels of kV and mAs and demonstrates that manipulation of either kV alone, mAs alone, or kV and mAs together are all possible means of optimizing radiation dose versus image quality. Acquisition parameters are as follows: top left – 120 kV, 206 mA; bottom left – 120 kV, 103 mAs; top right – 120 kV, 121 mAs; bottom right – 100 kV, 124 mAs

In theory, and in isolated practice, there are numerous combinations of CT acquisition parameters that may be chosen in the fine-tuning process to achieve the necessary image quality for diagnosis. (Fig. 1) There is less known, however, about how CT acquisition parameters vary in actual population-wide practice. Users adjust acquisition parameters at their own institutions and may make very different choices given the same inputs of patient body habitus and clinical indication even on the same machine make and model. Additionally, many CT scanners have automated systems to control acquisition settings (i.e. automatic exposure control [AEC]), making predicting what acquisition factors contribute to dose variation in clinical practice difficult since each CT vendor implements AEC differently. Relatively little is known regarding how radiology departments adjust acquisition parameters and the extent to which the variation in specific acquisition parameter contributes to observed CT dose variation.

This paper seeks to model the relationship between CT acquisition parameters and the radiation dose generated by each exam in actual clinical practice. Our hypothesis is that dose monitoring data can be used to firstly parameterize the impact of different acquisition parameters on patient dose, to secondly describe the frequency and magnitude of adjustment in patient dose over patient size and center-to-center, and to finally identify which acquisition parameters are most responsible for existing variation of radiation dose in current practice. Understanding current practices would inform future efforts toward dose optimization.

## Methods

### Data sources

This study was completed using data assembled in the University of California, San Francisco International CT Dose Registry. This dataset has been used and described in previous publications, as have the means by which it was collected [3, 9–11]. In short, the registry prospectively collected data from 162 diverse imaging facilities and hospitals from 7 countries including 20 US states, including academic, public, and private institutions, outpatient, emergency department, and in-patient settings, trauma centers, and cancer hospitals. Most of the data are from the US All contributing institutions used Radimetrics™ Radiation Dose Management Solution (Bayer HealthCare). The University of California, San Francisco Institutional Review Board approved the study, providing a waiver for individual informed consent, and collaborating institutions obtained IRB approval locally, or relied on University of California, San Francisco approval.

### CT examinations

All patients included in the study are 18 years or older. All CT exams included in the study were performed for the same indication—abdomen routine exams, not otherwise specified. The definition of this indication and the approach for identifying it has been previously described, validated, and shown to be 91% accurate [12]. The CT acquisition parameters considered as inputs in this study are the beam energy (kV), mAs, spiral pitch, the number of phases of each exam, and the scan length. In our analysis, we transformed pitch by taking its inverse to produce a positive correlation between pitch and radiation dose. The outcome of interest is the dose-length product (DLP) measured in milliGray-centimeters (mGy-cm). The dose and acquisition parameters were extracted from the Digital Imaging and Communications in Medicine (DICOM) metadata associated with each examination.

Exams with missing or erroneous values in radiation dose or any of the predictive acquisition parameters were removed from the study (*N* = 123,253, 9%). As a result, only 155 of the 162 imaging facilities and hospitals that contributed data to the registry were included in this study. The 7 (4% of total) excluded imaging facilities and hospitals consistently submitted incomplete data to the Registry (e.g., always deleting a single acquisition parameter).

### Modeling radiation dose as a function of CT acquisition parameters

The theoretical relationship between the DLP and the CT acquisition parameters considered in this study is that the DLP is proportional to each CT acquisition parameter, taken to some power [13, 14].

$$DLP=C\times k{V}^{{\beta }_{kV}}\times mA{s}^{{\beta }_{mAs}} \times \frac{1}{pitc{h}^{{\beta }_{pitch}}} \times scanlengt{h}^{{\beta }_{scanlength}} \times phas{e}^{{\beta }_{phase}}$$

where *C* is a constant, *kV, mAs, pitch, scanlength,* and *phase* are the values of each CT acquisition parameter, and ${\beta }_{X}$ is a power unique to CT acquisition parameter *X*. Existing theoretical understanding of the relationship between DLP and CT acquisition parameters indicates that we should expect ${\beta }_{kV}\approx 2.5$ and ${\beta }_{X}\approx 1$ for all other *X*. [14].

The equation above is not a linear combination but may be converted into one by log-transforming both sides. In other words, we equivalently parametrize the equation above using a linear mixed regression model with log(*DLP*) as the outcome, all CT acquisition parameters as predictors, and with the outcome and all predictors log-transformed. We add a random effect to account for the potential confounding impact of the scanner on which an examination is performed. The regression equation can be parametrized as follows:

$$\mathrm{log}\left(DLP\right)={\beta }_{0}+{\beta }_{kV}\mathrm{log}\left(kV\right)+{\beta }_{pitch}\mathrm{log}\left(\frac{1}{pitch}\right)+{\beta }_{scanlength}\mathrm{log}\left(scanlength\right)+{\beta }_{phase}\mathrm{log}\left(phase\right)+Z+\upepsilon$$

where ${\beta }_{0}=\mathrm{log}\left(C\right)$ and $Z\sim N\left(0,{\sigma }_{C}^{2}\right), \epsilon \sim N(0,{\sigma }_{R}^{2})$ are scanner random effect and residuals, with standard deviations ${\sigma }_{c},{\sigma }_{R}$, respectively. A comparison of the distribution of DLP values estimated by this model and the raw observed DLP can be found in figure A1.

Note that, as a result of the logarithmic transformations made therein, this model allows for the percent increase in DLP induced by a percent increase in a CT acquisition parameter to not be dependent on the initial value from which the percent increase in the CT acquisition parameter is made. In other words, this model allows for the assessment that, for every X% increase in the acquisition parameter (e.g., mAs), the radiation dose (DLP) increases by Y%. Consequently, interpretations of this model’s results can be presented in terms of percent increases in the CT acquisition parameter (such as “10% increase” or “geometric standard deviation increase”) rather than raw increases in the CT acquisition parameter (such as “10 mAs increase” or “arithmetic standard deviation increase”), allowing us to disregard the units in which the CT acquisition parameters are measured. Note that, for ${\beta }_{P}>1$, a modest percent increase in the parameter *P* will result in a comparatively large increase in DLP.

To interpret the results of this model, the “impact” of each CT acquisition parameter on the DLP will be presented in one of three conceptualizations, holding all other parameters constant:

1. What is the percent increase in DLP when the parameter is increased by 10%??
2. What is the percent increase in DLP when the parameter is increased by 15%?
3. What is the percent increase in DLP when the parameter is multiplied by one geometric standard deviation (which this paper will describe as “increasing by one standard deviation”)?

Assessing the odds ratio of an event per non-unit increase in risk factor is well documented [15, 16]. The difference between the three conceptualizations presented is that (3), unlike (1) and (2), will change depending on the distribution of acquisition parameters in the general population. Thus, the assessment of (1) and (2) illustrate the raw (theoretical) relationship between the DLP and CT acquisition parameters; for a CT acquisition parameter linearly associated with dose (like mAs), we expect an increase in DLP of 10% per increase in the parameter of 10%. Conversely, the assessment of (3) captures how much this relationship manifests in the variation of DLP in common practice. For example, a CT acquisition parameter which, in practice, is rarely manipulated or manipulated only slightly may be of minimal impact in the assessment of (3), even if it were highly impactful in (1) and (2). We calculate, for each of the three conceptualizations of an increase in acquisition parameters the resulting percent change in the DLP. The goal is to identify which parameters have a strong theoretical relationship with DLP, and which parameters actually explain the variation of DLP in real-world practice. Changes of 10% and 15% were selected as small, reasonable theoretical changes to make in CT acquisition parameters for a hospital pursuing radiation dose reduction.

We graphically demonstrate how an increase of 10%, 15%, and one standard deviation in each parameter influences the DLP. For each figure, we plot a random sample of 5000 data points as the full sample size of over 1 million exams makes the figures difficult to interpret. The data points are jittered to better see the relationships (because so many patients have the same acquisition parameter values).

All statistical analyses performed in this paper were done by the lead author using R ver 4.2.1. Package lme4 ver 1.1–29 was used for mixed regression model fitting.

## Results

A total of 1,276,974 routine abdomen CT exams performed on patients 18 years of age or older at 155 imaging facilities or hospitals between November 1, 2015, and Jan 1, 2021, are included. This reflects exams obtained on 523 individual CT scanners representing 103 scanner models made by the 4 largest CT manufacturers (Table 1).

**Table 1:** CT examinations included in this report. Age percentages add up to 99% due to rounding

	*N*	%
Total sample size	1,276,974	100
Patient characteristics		
Male	566,836	44
Female	707,341	55
Non-binary or unknown	2,797	1
Age		
Age 18–20	21,735	2
Age 21–30	105,728	8
Age 31–40	141,254	11
Age 41–50	182,345	14
Age 51–60	244,302	19
Age 61–70	258,332	20
Age 71–80	184,187	15
Age 81 +	129,091	10
Exam characteristics		
Canon	121,559	10
General Electric	533,056	42
Philips	220,608	17
Siemens	401,751	31
Location		
United States	1,118,236	88
Europe	76,471	6
Israel	65,841	5
Japan	16,426	1

The descriptive statistics of each parameter are shown in Table 2. There was relatively little change in kV compared to other CT acquisition parameters, as seen by the comparatively narrow range in percentile values. Automated kV selection does not seem to be commonly applied. Indeed, for over 80% of exams in this study, the kV was equal to 120. For 58% of imaging facilities or hospitals and 62% of scanners, more than 90% of exams had kV equal to 120. The full range of observed kV values (70 to 150) and the large variation of DLP associated with this range, suggest that in practice, kV is not manipulated to its full potential.

**Table 2:** Distribution of CT acquisition parameter values

	DLP (mGy-cm)	kV	mAs	Spiral Pitch	Number of Phases	Scan Length (mm)
Geometric mean	666	118	154	1	1	414
Geometric standard deviation	2.02	1.07	1.70	1.28	1.57	1.52
1^st^ Percentile	135	100	41	0.55	1	105
5^th^ Percentile	237	100	59	0.61	1	182
25^th^ Percentile	423	120	107	0.81	1	355
50^th^ Percentile	659	120	165	0.98	1	477
75^th^ Percentile	1062	120	230	1.19	2	533
95^th^ Percentile	2047	120	327	1.38	3	613
99^th^ Percentile	3261	140	445	1.50	5	709

The three conceptualizations of “impact” of the acquisition parameters on DLP are provided in Table 3. With respect to the theoretical relationship between CT acquisition parameters and the DLP (reflecting conceptualizations 1 and 2), the greatest association is found in kV—a 10% increase in kV is associated with a 29% increase in the DLP. The same magnitude (10%) increases in mA, (inverse) spiral pitch, number of phases, and scan length are associated with far smaller increases in the DLP, ranging from 5 to 9% across the different parameters. A 15% increase in kV is associated with a 45% increase in the DLP, while the same magnitude increases in other parameters are associated with an increase of 7–14% in the DLP.

**Table 3:** Impact of changes in the CT acquisition parameters on the dose length product

	kV	mAs	(Inverse) Spiral Pitch	Number of Phases	Scan Length (mm)
DLP Increase per 10% Increase in acquisition parameter	29%	9%	8%	8%	5%
DLP Increase per 15% Increase in acquisition parameter	45%	14%	12%	12%	7%
DLP Increase per geometric standard deviation increase in acquisition parameters	20%	64%	22%	45%	24%

The results are different for the impact of a one standard deviation increase in the acquisition parameters. An increase of one standard deviation (7% increase) of kV is associated with only a 20% increase in DLP, whereas an increase of one standard deviation (70% increase) in mAs is associated with a far greater 64% increase in DLP. An increase in one standard deviation in the inverse spiral pitch (28% increase), number of phases (57% increase), and scan length (52% increase) result in 22–45% increases in DLP. This reflects the more common manipulation of acquisition parameters other than kV in actual practice, resulting in a greater impact on the DLP.

### Graphical visualization of the impact of increases in acquisition parameters on DLP

**Fig. 2:**
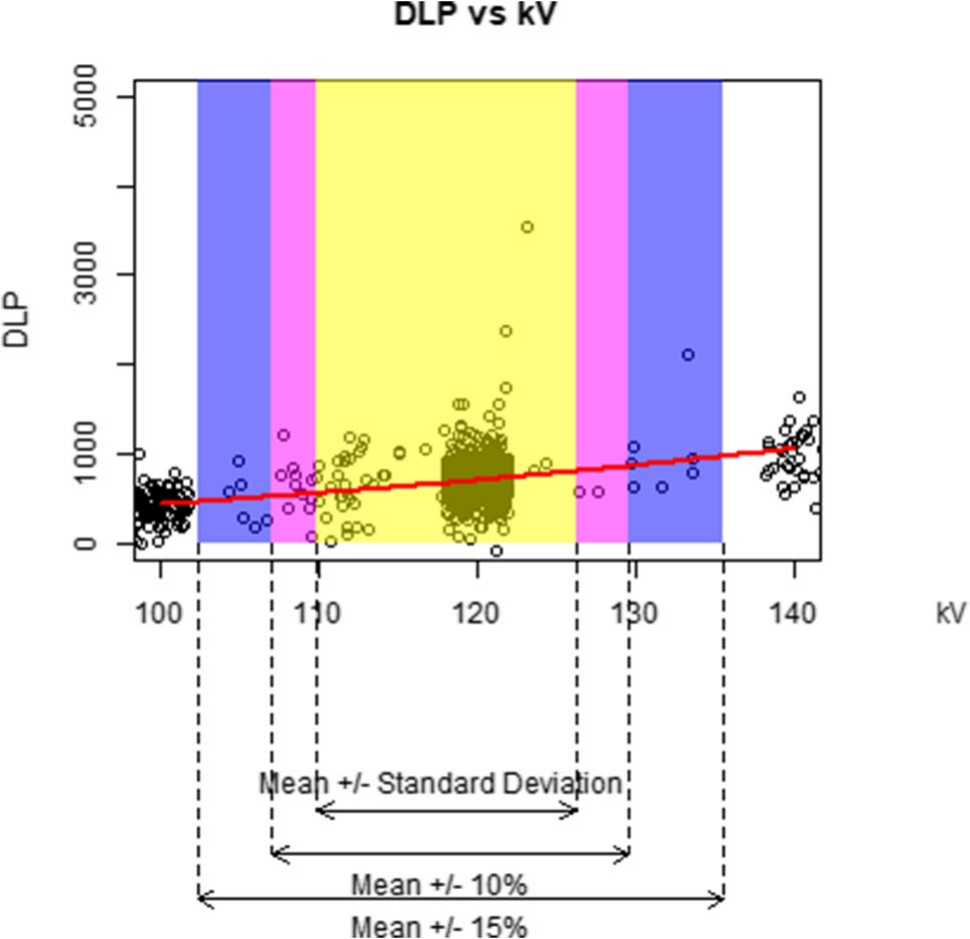
Visualization of the impact of increases to kV on DLP

**Fig. 3:**
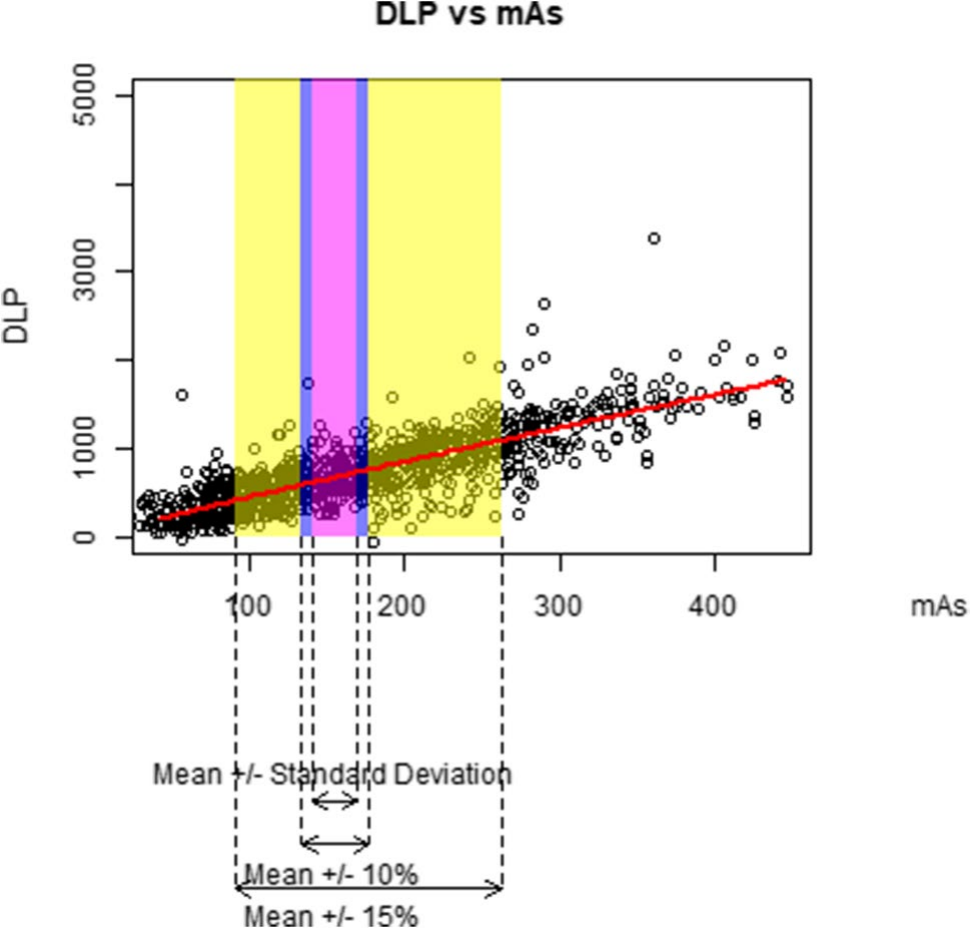
Visualization of the impact of increases to mAs on DLP

**Fig. 4:**
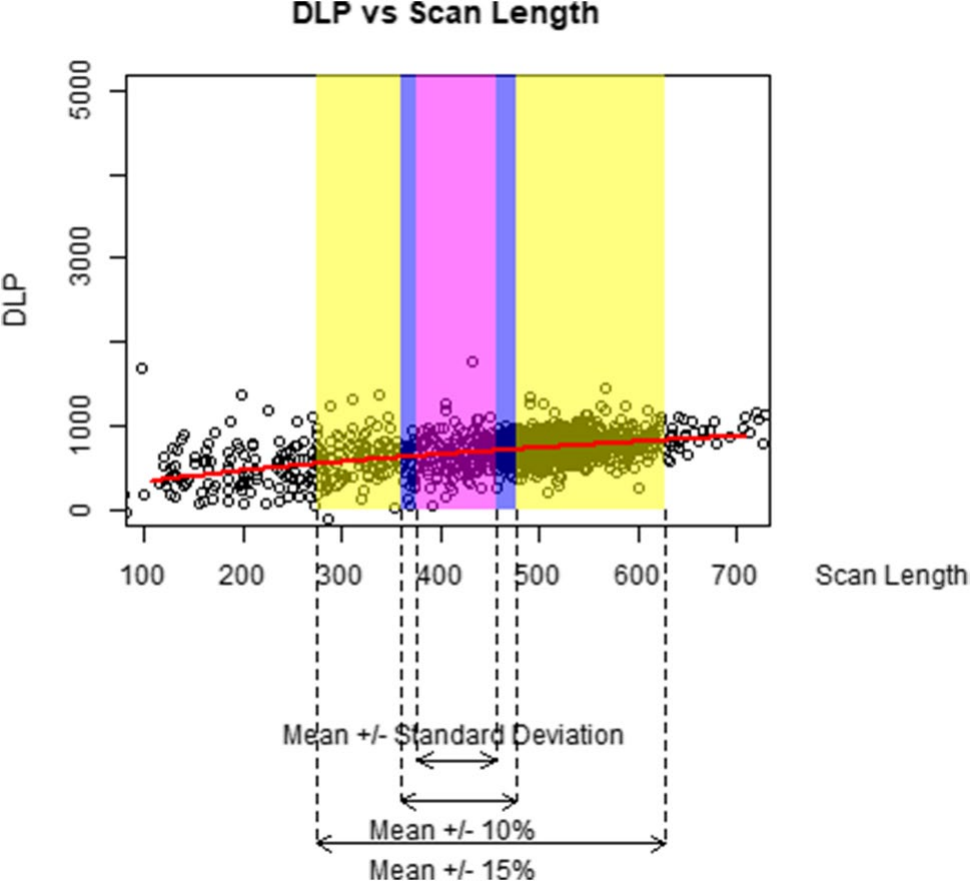
Visualization of the impact of increases to scan length on DLP

**Fig. 5:**
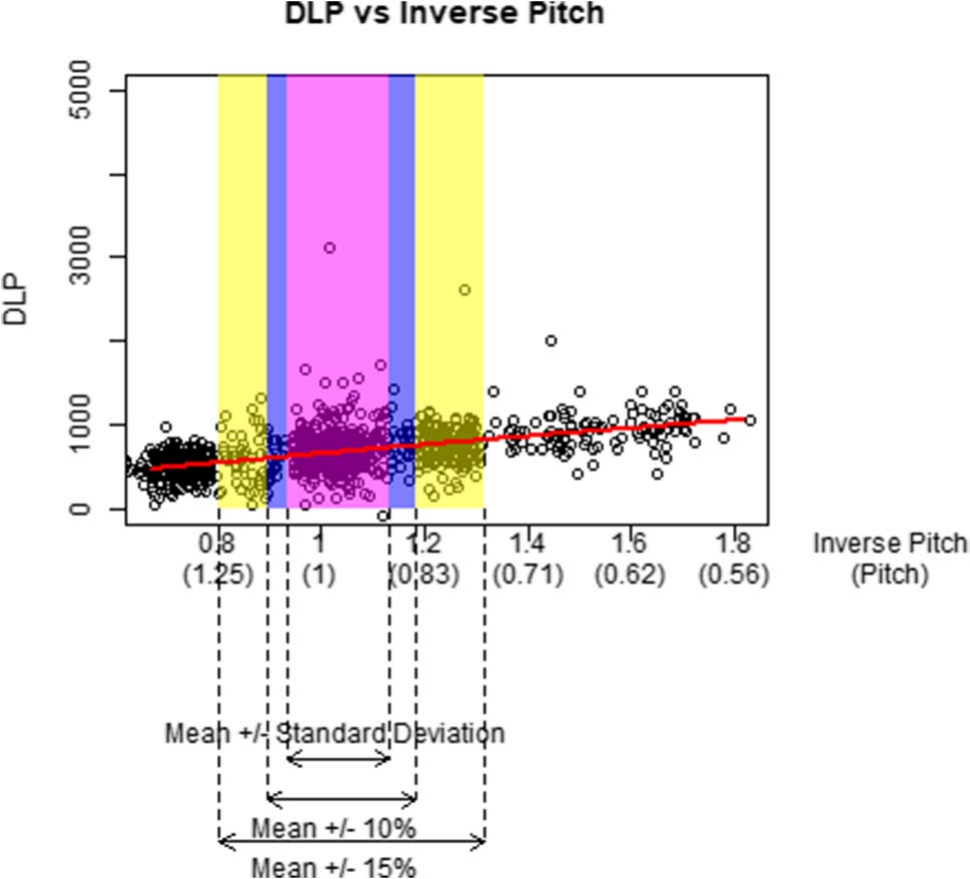
Visualization of the impact of increases to inverse spiral pitch on DLP

**Fig. 6:**
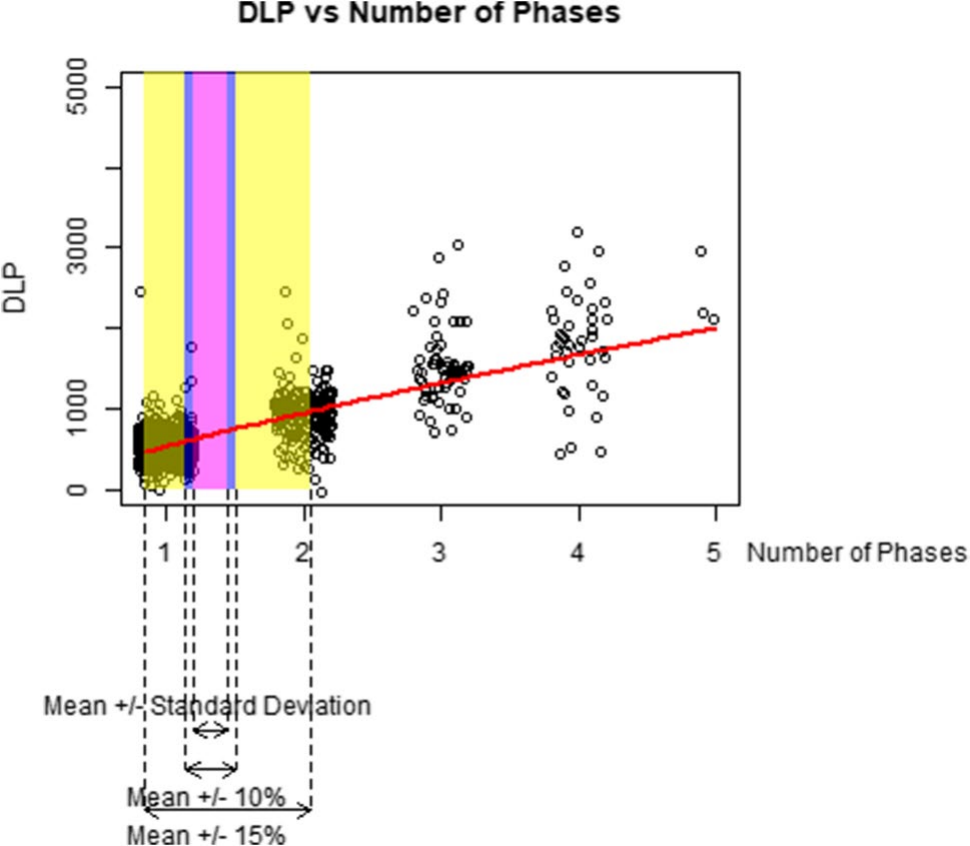
Visualization of the impact of increases to the number of phases on DLP

Figures 2, 3, 4, 5, and 6 graphically display the relationship between the DLP and each acquisition parameter. On each figure, the scattered points show a random sample of 5000 jittered observed examination values and the red line indicates the modelled relationship between the DLP and the studied acquisition parameter when all other CT acquisition parameters are controlled; this model is based on all 1.3 million exams in the study, not only the 5000 scattered points. The purple box identifies exams where the acquisition parameter is within 10% of the geometric mean, the blue box within 15% of the geometric mean, and the yellow box exams within one geometric standard deviation of the geometric mean. Note that, while the regression models used in this paper involve a collection of log-transformations, the x-axis and y-axis of Figs. 2, 3, 4, 5, and 6 are all presented on a linear scale for interpretability.

For kV (Fig. 2), the 10% box (purple) ranges from 107 to 130, reflecting expected DLP values ranging from 514 to 857 mGy-cm, accounting for 85% of all exams. The 15% box (blue) ranges from 103 to 136 kV, reflecting expected DLP ranging from 456 to 967 mGy-cm, accounting for 86% of all exams. The one standard deviation box (yellow) ranges from 110 to 126 kV, narrower than the 10% values, and reflecting expected DLP ranging from 554 to 800 mGy-cm, accounting for 84% of all exams.

For mAs (Fig. 3), there is a wider range of observed values, such that the standard deviation is far wider than the 10% or 15% deviation. The 10% box ranges from 140 to 169 mAs, reflecting expected DLP ranging from 608 to 725 mGy-cm, reflecting 12% of all exams. The 15% box ranges from 134 to 177 mAs, reflecting expected DLP ranging from 583 to 756 mGy-cm, reflecting 18% of all exams. The one standard deviation box ranges from 90 to 262 mAs, reflecting expected DLP ranging from 405 to 1086 mGy-cm, reflecting 68% of all exams.

Note that within the confines of the purple and blue boxes, the kV induces the greatest change in expected DLP among all acquisition parameters, indicating a strong impact of kV on DLP based on the theoretical opportunity if kV were manipulated. In contrast, within the confines of the yellow box indicating actual practice, kV shows a more modest impact on the dose in comparison to the other parameters.

The impact of inverse spiral pitch, number of phases, and scan length (Figs. 4, 5, and 6) are similar to mAs in that an increase of one standard deviation in any of these CT acquisition parameters (yellow box) is associated with a greater increase in DLP than an increase of one standard deviation (7% increase) in kV. This is despite the fact that, like mAs, the theoretical relationship between DLP and these CT acquisition parameters (purple and blue boxes) is more modest than the one between DLP and kV.

## Discussion

Our study confirms the well-known relationships between CT acquisition parameters and patient radiation exposure when observed in clinical patient scans on a large scale. The CT acquisition parameters mAs, inverse spiral pitch, number of phases, and average scan length are all expected to have a linear relationship with DLP when other acquisition parameters are fixed [14]. Indeed, we observe most to have a close to a linear relationship with DLP (i.e., a 10% or 15% increase in parameter values results in, respectively, a close to 10% or 15% increase in DLP). A percent increase in beam energy should reflect an equivalent increase in DLP, taken roughly to the power of 2.5 [13]. By this rule, a 15% increase in kV (reflecting an increase from 120 to 138 kV) would increase the DLP by 42%. Our data closely match this (Table 2). Our data shows scan length having a weaker than linear relationship with DLP, a deviation from established understanding. For every 10% increase in scan length, we observed a 5% increase in DLP (Table 2).

While our data confirmed the most well-established relationships between radiation dose and CT acquisition parameters, it also demonstrated large differences in how frequently each CT acquisition parameter is manipulated. We clearly see our community adopting tube current (i.e., mAs) modulation, but most sites do not follow well-established optimal beam energy recommendations that adjust beam energy with patient size.

To fully optimize radiation dose, one must have a willingness to manipulate kV. It is well-established that reducing beam energy can reduce radiation dose with equal to even improved image quality from a theoretical standpoint and in clinical practice, even when coupled with accompanying manipulation of mAs [17–20]. Simply stated, phantom-based and clinical research has demonstrated optimal beam energies should increase with patient size and decrease for a fixed patient size when the signal from Iodine is of clinical diagnostic importance [17–20]. Unfortunately, our study shows kV to have a very low standard deviation in actual practice, meaning that manipulation of kV remains largely underutilized, with or without accompanying manipulation of other acquisition parameters. The range of kV values observed in our study is grossly deviant from values theoretically studied in established literature. No examinations were observed with kV lower than 100, despite values of 70–80 kV being suggested as optimal for very small patients; similarly, less than 1% of examinations were observed with kV of 140, a value suggested as optimal for very large patients [17, 18, 21]. Although automated adjustment functions for kV have been commercialized relatively recently compared to analogous functions for mAs, the extreme nature of observed kV distributions in the population seems to indicate that, even when such functions are available, they are not being used. We therefore must conclude that, while the community has adopted mAs modulation, the use of kV values outside of the most common 120 kV remains an untapped area of CT dose and image quality optimization potential.

One limitation of this study is a lack of comparison between observed radiation doses and a measure of image quality, such as image noise. While this does not affect our conclusions on how CT acquisition parameters affect radiation dose, we do nonetheless encourage adjustments to CT acquisition parameters to be made with image quality considerations in mind. As for the specific CT examinations presented in this paper, they represent radiological practice performed in the context of a collaborative project to reduce radiation dose. In this project, surveys were conducted periodically to gauge whether radiologists, technicians, and other relevant personnel objected to the impacts of dose reduction on image quality; surveyed parties had no objections to raise [9]. Thus, at least for the typical examination presented in this paper, image quality seemed to be adequate.

This study was performed on adult CT examinations only. The degree to which its results can be generalized to children, and the additional statistical considerations that may need to be made to attend to children’s data, is a topic of future study.

CT vendors could take note of our results and do more to make their AEC systems easier to implement both tube current and beam energy modulation because in current practice beam energy modulation is rarely occurring. Professional societies could reinforce education on optimal CT acquisition parameters to enhance the frequency and magnitude of beam energy modulation. If a reaction to our work includes more sites implementing beam energy adjustments that are iodine task and patient size specific, we can be confident based on prior research image quality and or dose optimization will improve.

### Supplementary Information

Below is the link to the electronic supplementary material.

Supplementary file1 (PDF 124 KB)

## Abbreviations

AEC: Automatic exposure control
ALARA: As low as reasonably achievable
CT: Computed tomography
CTDI_vol_: Volume CT dose index
DICOM: Digital Imaging and Communications in Medicine
DLP: Dose-length product
IRB: Institutional review board
kV: Beam energy (kilovoltage)
mAs: Milliamperage seconds
SD: Standard deviation

## Declarations

### Guarantor

The scientific guarantor of this publication is Yifei Wang.

### Statistics and biometry

Yifei Wang kindly provided statistical advice for this manuscript.

### Study subjects or cohorts overlap

This study was completed using data assembled in the University of California, San Francisco International CT Dose Registry, previously described in several papers, including:

Smith-Bindman R, Wang Y, Chu P, Chung R, Einstein AJ, Balcombe J, Cocker M, Das M, Delman BN, Flynn M, Gould R, Lee RK, Yellen-Nelson T, Schindera S, Seibert A, Starkey J, Suntharalingam S, Wetter A, Wildberger JE, Miglioretti DL. International variation in radiation dose for computed tomography examinations: prospective cohort study. *BMJ* (Clinical research ed) 2019;364:k4931. https://doi.org/10.1136/bmj.k4931

Smith-Bindman R, Chu P, Wang Y, Chung R, Lopez-Solano N, Einstein AJ, Solberg L, Cervantes LF, Yellen-Nelson T, Boswell W, Delman BN, Duong PA, Goode AR, Kasraie N, Lee RK, Neill R, Pahwa A, Pike P, Roehm J, Schindera S, Starkey J, Suntharalingam S, Jeukens C, Miglioretti DL. Comparison of the Effectiveness of Single-Component and Multicomponent Interventions for Reducing Radiation Doses in Patients Undergoing Computed Tomography: A Randomized Clinical Trial. JAMA internal medicine 2020; *JAMA Intern Med*. 2020;180(5):666–675. https://doi.org/10.1001/jamainternmed.2020.0064

Chu PW, Yu S, Wang Y, Seibert JA, Cervantes LF, Kasraie N, Chu CA, Smith-Bindman R. Reference phantom selection in pediatric computed tomography using data from a large, multicenter registry. *Pediatric Radiology* 2022;52(3):445–452. https://doi.org/10.1007/s00247-021-05227-0

Bos D, Yu S, Luong J, Chu P, Wang Y, Einstein AJ, Starkey J, Delman BN, Duong PT, Das M, Schindera S, Goode AR, MacLeod F, Wetter A, Neill R, Lee RK, Roehm J, Seibert JA, Cervantes LF, Kasraie N, Pike P, Pahwa A, Jeukens CRLPN, Smith-Bindman R. Diagnostic reference levels and median doses for common clinical indications of CT: findings from an international registry. *Eur Radiol*. 2021 Oct 13. https://doi.org/10.1007/s00330-021-08266-1. Epub ahead of print. PMID: 34642811.

These previously published studies featured most or all patients featured in our study. Subject matter of these previously published studies, however, are unrelated to the manipulation of CT acquisition parameters and their impact on observed radiation dose distributions, which is the subject of our study.

### Methodology

• prospective

• observational

• multicenter study

## References

[1] Kanal KM, Butler PF, Sengupta D, Bhargavan-Chatfield M, Coombs LP, Morin RLUS (2017). Diagnostic reference levels and achievable doses for 10 adult CT examinations. Radiology.

[2] Kanal KM, Butler PF, Chatfield MB (2022). U.S. Diagnostic reference levels and achievable doses for 10 pediatric CT examinations. Radiology.

[3] Smith-Bindman R, Wang Y, Chu P (2019). International variation in radiation dose for computed tomography examinations: prospective cohort study. BMJ (Clin Res ed).

[4] Frija G, Damilakis J, Paulo G, Loose R, Vano E, European Society of Radiology (ESR) (2021). Cumulative effective dose from recurrent CT examinations in Europe: proposal for clinical guidance based on an ESR EuroSafe Imaging survey. Eur Radiol..

[5] Berrington de Gonzalez A, Mahesh M, Kim KP (2009). Projected cancer risks from computed tomographic scans performed in the United States in 2007. Arch Intern Med.

[6] Loose RW, Vano E, Mildenberger P (2021). Radiation dose management systems-requirements and recommendations for users from the ESR EuroSafe Imaging initiative. Eur Radiol..

[7] National Electrical Manufacturers Association (NEMA) (2019). Computed Tomography Dose Check NEMA XR 25–2019. ID 100037. 2019. https://www.nema.org/Standards/view/Computed-Tomography-Dose-Check

[8] Solberg LI, Wang Y, Whitebird R, Lopez-Solano N, Smith-Bindman R (2020). Organizational factors and quality improvement strategies associated with lower radiation dose from CT examinations. J Am Coll Radiol.

[9] Smith-Bindman R, Chu P, Wang Y (2020). Comparison of the effectiveness of single-component and multicomponent interventions for reducing radiation doses in patients undergoing computed tomography: a randomized clinical trial. JAMA Intern Med..

[10] Chu P, Yu S, Wang Y (2022). Reference phantom selection in pediatric computed tomography using data from a large, multicenter registry. Pediatr Radiol.

[11] Bos D, Yu S, Luong J (2021). Diagnostic reference levels and median doses for common clinical indications of CT: findings from an international registry. Eur Radiol..

[12] Smith-Bindman R, Yu S, Wang Y (2022). An Image Quality-informed Framework for CT Characterization. Radiology.

[13] Hsieh J (2003). Analytical models for multi-slice helical CT performance parameters. Med Phys.

[14] Hsieh J (2015). Computed tomography: principles, design, artifacts, and recent advances.

[15] Kaptoge S, Di Angelantonio E, Lowe G (2010). C-reactive protein concentration and risk of coronary heart disease, stroke, and mortality: an individual participant meta-analysis. Lancet..

[16] Wensley F, Gao P, Burgess S (2011). Association between C reactive protein and coronary heart disease: mendelian randomisation analysis based on individual participant data. BMJ..

[17] Kalender WA, Deak P, Kellermeier M, van Straten M, Vollmar SV (2009). Application-and patient size-dependent optimization of x-ray spectra for CT. Med Phys.

[18] Yu L, Bruesewitz MR, Thomas KB, Fletcher JG, Kofler JM, McCollough CH (2011). Optimal tube potential for radiation dose reduction in pediatric CT: principles, clinical implementations, and pitfalls. Radiographics.

[19] Niemann T, Henry S, Faivre JB (2013). Clinical evaluation of automatic tube voltage selection in chest CT angiography. Eur Radiol.

[20] Raman S, Johnson PT, Deshmukh S, Mahesh M, Grant KL, Fishman EK (2013). CT dose reduction applications: available tools on the latest generation of CT scanners. J Am Coll Radiol.

[21] Yu L, Li H, Fletcher JG, McCollough CH (2010). Automatic selection of tube potential for radiation dose reduction in CT: a general strategy. Med Phys.

