# Supplementary material for: CT acquisition parameter selection in the real world: impacts on radiation dose and variation amongst 155 institutions
Source: Eur Radiol. 2023 Aug 30;34(3):1605–13. doi: 10.1007/s00330-023-10161-w (PMC10873435; doi:10.1007/s00330-023-10161-w)

Table A1 – Impact of changes in the CT acquisition parameters on the dose length product, based on percent increases equivalent to one geometric standard deviation increase of each CT acquisition parameters

	kV	mAs	(Inverse) Spiral Pitch	Number of Phases	Scan Length (mm)
DLP Increase per 7% (kV gSD) Increase in acquisition parameter	20%	6%	6%	6%	4%
DLP Increase per 70% (mAs gSD) Increase in acquisition parameter	315%	64%	54%	55%	31%
DLP Increase per 28% (inverse spiral pitch gSD) Increase in acquisition parameter	94%	26%	22%	23%	14%
DLP Increase per 57% (number of phases gSD) Increase in acquisition parameter	235%	52%	44%	45%	26%
DLP Increase per 52% (scan length gSD) Increase in acquisition parameter	207%	47%	41%	41%	24%

Figure A1 – Distribution of observed dose-length product (mGy-cm), compared to dose-length product, as estimated by our regression model

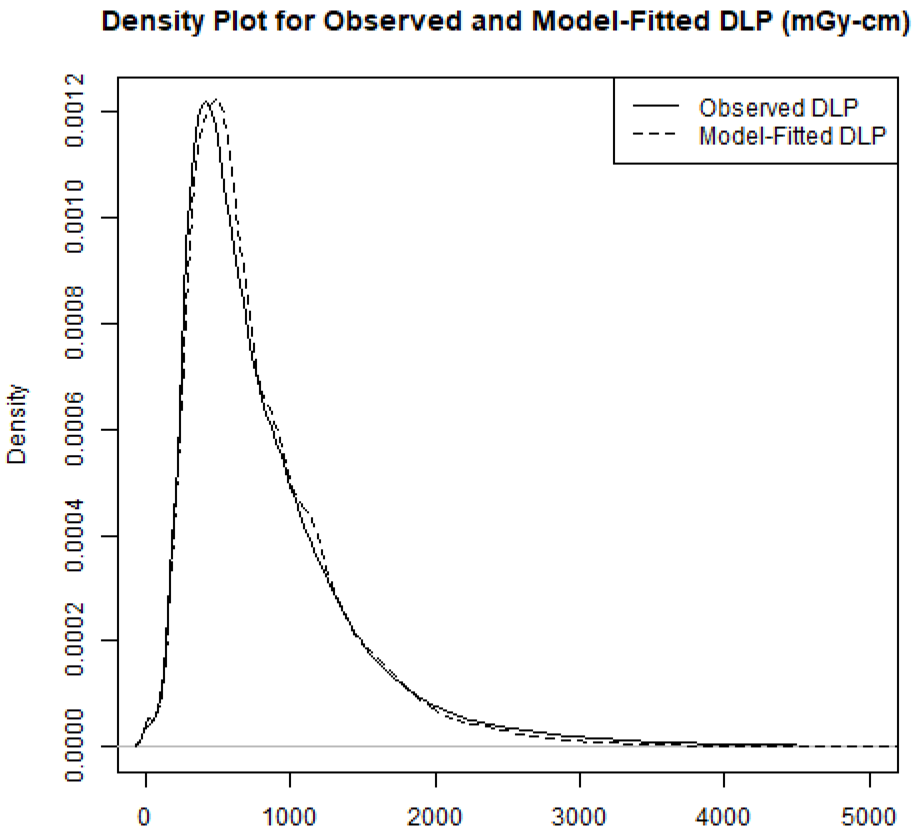

Supplement: Supplementary file 1 — Supplementary file1 (PDF 124 KB) [file 330_2023_10161_MOESM1_ESM.pdf]
